# A miR‐15a related polymorphism affects NSCLC prognosis via altering ERCC1 repair to platinum‐based chemotherapy

**DOI:** 10.1111/jcmm.17566

**Published:** 2022-09-30

**Authors:** Ping Xue, Guopei Zhang, Hongchao Zhang, Su Cui, Liang Zhang, Tao Yu, Mingyang Xiao, Liuli Li, Xiaobo Lu

**Affiliations:** ^1^ Department of Toxicology, School of Public Health China Medical University Shenyang China; ^2^ Department of Thoracic Surgery Ward 2 The First Hospital of China Medical University Shenyang China; ^3^ Department of Thoracic Surgery Liaoning Cancer Hospital & Institute Shenyang China

**Keywords:** *ERCC1*, miR‐15a, non‐small cell lung cancer, platinum‐based chemotherapy, polymorphism

## Abstract

Platinum‐based chemotherapy is regarded as a preferential curative‐intent option for non‐small cell lung cancer (NSCLC), while the acquired drug resistance has become a major obstacle that limits its clinical application. Since the repair efficiency of tumour cells to platinum‐DNA adducts plays a crucial role in chemotherapy resistance, we aimed to explore whether several meaningful polymorphisms of DNA repair genes were associated with the benefits of platinum‐based chemotherapy in NSCLC patients. Firstly, six single nucleotide polymorphisms (SNPs) located in the 3'untranslated region (3'UTR) of three DNA repair genes were detected in 246 NSCLC patients receiving platinum‐based chemotherapy and analysed the correlation of these candidate SNPs with the overall survival. Cox proportional hazard model showed that NSCLC patients carrying ERCC1 rs3212986 AA genotype had a shorter overall survival compared to those with CC. Mechanistically, we performed tumour chemosensitivity assay to observe the convincing linkage of rs3212986 polymorphism with ERCC1 expression and cisplatin sensitivity. The subsequent in vitro experiments identified that rs3212986 polymorphism altered the post‐transcriptional regulation of ERCC1 via affecting the binding of miR‐15a, and further changed the sensitivity to platinum analogue. It reminded that patients carrying *ERCC1* rs3212986 CC homozygote were expected to respond better to platinum‐based chemotherapy due to a lower expression of ERCC1. Compared with previous studies, our current comprehensive study suggested that rs3212986, a 3'UTR polymorphism in *ERCC1*, might have clinical relevance in predicting the prognosis of NSCLC patients receiving platinum‐based chemotherapy.

## 1 INTRODUCTION

Non‐small cell lung cancer (NSCLC) remains the leading cause of cancer‐related mortality worldwide,^1^ and its incidence and mortality in China have increased rapidly in the last two decades.^2^ Approximately 50% of NSCLC patients undergoing adjuvant chemotherapy will relapse within 5 years,^3^ because only a minority of them responded better to the standard treatments including chemotherapy.^4^ Accordingly, there is an urgent need to identify reliable prognostic biomarkers to assist in developing personalized therapies.

Although platinum‐based chemotherapy has been determined as a standard first‐line treatment for advanced NSCLC, the frequent drug resistance remains a major obstacle that limits the therapeutic efficacy in clinic.^5^ Platinum‐DNA adduct accumulation is a determinant step for the cytotoxicity of platinum‐based antitumor agents which lead to the destabilization of double helix, blocking replication and inhibiting transcription.^6^ However, DNA repair capacity, which varies widely among individuals, plays a fundamental role in timely removing DNA adducts.^7^ In general, a high DNA repair capacity of tumour cells is a warning of potential chemotherapy resistance of NSCLC patients.^8^ As known, there are at least several DNA repair systems in the human body, such as nucleotide excision repair (NER), base excision repair (BER) and mismatch repair (MMR).^9^ Among them, NER is the most significant and flexible one for excising platinum‐DNA adducts.^10^ The main repair steps of NER include the separation of double helix at lesion sites, the excision of the lesion‐containing single stranded DNA fragment, the synthesis of a new DNA fragment to replace the gap and the ligation of the remaining single‐stranded nick.^11^ Therefore, the NER pathway plays a pivotal role in repairing platinum‐DNA adducts and may affect the sensitivity of individuals to platinum chemotherapy potentially.^12^, ^13^

The excision repair cross‐complementation group 1 (*ERCC1*) is a known gene as a rate‐limiting enzyme in the NER pathway,^14^ and emerging evidence suggested that ERCC1 expression acted as a promising biomarker to predict the prognosis of multiple cancers.^15^ *ERCC1* overexpression showed a certain clinical resistance to platinum‐based chemotherapy in ovarian, cervical, colorectal carcinomas and NSCLC.^16^, ^17^, ^18^, ^19^ Additionally, NSCLC patients with complete resection of ERCC1‐negative tumours benefited more from cisplatin‐based adjuvant chemotherapy than those with ERCC1 positive tumours.^20^ In other words, a higher expression of ERCC1 may indicate a potential failure in chemotherapy due to the efficient repair of genetic damage in tumour cells induced by platinum analogues. Although the clinical trial of customized chemotherapy relying on ERCC1 expression has been carried out.^21^ Actually, it is often difficult to obtain sufficient tumour tissues to quantify the expression of ERCC1 clinically. Instead, the germline polymorphisms are easy to measure and constant over time and have gradually become promising biomarkers to predict clinical outcomes of NSCLC patients. Single nucleotide polymorphisms (SNPs) in human DNA repair genes have been reported to modulate DNA repair capacity, which is generally considered as valid biomarkers to reflect the repair efficiency of chemical‐induced DNA damage.^22^ Notably, the variation of coding regions can affect the function of protein, while other variations of non‐coding regions may affect gene expression and protein activity. Several studies revealed that the SNPs of rs1007616, rs735482 and rs3212986 located in the 3′ untranslated region (UTR) of *ERCC1* could reduce the stability of its mRNA and further affect DNA repair capacity,^23^, ^24^ which reminded us that the post‐transcriptional regulation of *ERCC1* might play a crucial role in the development of lung cancer.^25^, ^26^ MicroRNAs (miRNAs) are a kind of endogenous non‐coding RNA with the length of about 22 nucleotides, regulating the expression of genes by base paring with the 3'UTR of target mRNA generally.^27^ As known, the miRNAs play a growing important role in various essential and important biological processes, such as cell development, proliferation differentiation, apoptosis, signal transduction, viral infection and so on.^28^, ^29^ Therefore, the relationship between the allele‐specific alterations related to miRNAs and the sensitivity to platinum‐based chemotherapy in NSCLC patients needs to be investigated.

In the present study, a survival analysis of NSCLC patients received platinum‐based chemotherapy was firstly performed to evaluate the association of six candidate 3'UTR polymorphisms in DNA repair genes with the prognosis. Then, we found that ERCC1 rs3212986 polymorphism exhibited a more convincing linkage with the overall survival in the clinical investigation. Furthermore, NSCLC tumour tissue samples were detected to confirm the relationship between ERCC1 rs3212986 polymorphism and cisplatin sensitivity. Finally, a series of in vitro functional experiments were carried out to clarify whether the target polymorphism was causally linked with the sensitivity to platinum analogues via altering the post‐transcriptional regulation of ERCC1 due to a certain miRNA. Accordingly, our current comprehensive study contributes to identify the potential biomarkers in predicting the prognosis of NSCLC patients receiving curative‐intent chemotherapy.

## 2 MATERIALS AND METHODS

### 2.1 Study subjects and sample collection

In this study, we recruited 246 patients who had been histologically confirmed with NSCLC and received curative‐intent therapy including surgical resection and platinum‐based chemotherapy at the First Hospital of China Medical University from September 2013 to February 2015, and the inclusion criteria mainly included the following aspects: (1). NSCLC patients who have received curative‐intent therapy including surgical resection and platinum‐based chemotherapy (including common platinum analogues such as cisplatin [CDDP], carboplatin, nedaplatin, oxaliplatin and lobaplatin); (2). Follow‐up information was intact and reliable; (3). Other serious lung diseases were ruled out; (4). Sample quality was qualified; (5). Causes of death were predominantly due to lung cancer. Besides, all the subjects were Han people inhabiting in the northeast of China. Prior to this study, the protocol and consent form were approved by the Institutional Review Board of China Medical University and informed consent was obtained from each participant after detailing the purpose of this study. Afterwards, their demographic data including age, sex, career, smoking and drinking status were accurately recorded in questionnaires and the clinical information including tumour size, lymphatic metastasis, distant metastasis and Tumour‐Node‐Metastasis(TNM) staging was assessed by two veteran oncologists according to the criteria set by World Health Organization (Patient information was provided as a supplementary sheet in .xlsx format.). Besides, 5 ml blood samples were drawn from each participant and their fresh tumour tissues were collected during surgery, then immediately transferred into liquid nitrogen. Finally, our accumulation was stopped in February 2019 to guarantee a minimum follow‐up time of 4 years and the detailed follow‐up information was recorded. Overall survival (OS) was specified as the primary endpoint of this study, which was calculated from diagnosis to the last follow‐up or death due to any cause.

### 2.2 DNA extraction and TaqMan® Genotyping Assay

Genomic DNA was extracted with DNAzol Reagent (Invitrogen, USA) according to the manufacturer's protocol. Meanwhile, the DNA concentration and purity were determined by NanoDrop OneC Microvolume UV–Vis Spectrophotometer (Thermo Scientific™, USA) and the values of A260/A280 or A260/A230 of 1.8–2.2 proved the reliability of DNA quality. Subsequently, TaqMan® Genotyping Assay was performed to detect *ERCC1* rs3212986, rs735482, rs2336219, rs1007616, *MLH3* (mutL homologue 3) rs108621 and *hOGG1*(8‐oxoguanine DNA glycosylase) rs1052133. The samples were analysed with Genotyping Assay Reagents (ABI, USA, Stagapore) in the LightCycler 480 Real‐time PCR system (Roche, USA). The PCR reaction was running in a 20 μl reaction mixture, consisting of 10 μl of probe Mix, 5 μl (1×) of assay mix and 2 μl of DNA (25 ng/μl). Briefly, the PCR reaction program included an initial step at 95°C for 10 min, 40 cycles of denaturation at 95°C for 10 s, annealing/extension at 60°C for 1 min and finally chilling at 40°C for 30 s. To realize a phenotype‐blind genotyping process, the collection of data, the test of samples and the analysis of results were conducted by different experimenters, respectively. For rs1007616 and rs108621, thymine (T) was replaced with cytosine (C); cytosine (C) was replaced with adenine (A) in rs735482 and rs3212986; guanine (G) was replaced with adenine (A) in rs23362196; whereas in rs1052133 cytosine (C) was replaced with guanine (G). TaqMan SNP genotyping IDs were as follows: *ERCC1* (rs3212986, C>A, assay ID is C_2532948_10; rs735482, A>C, assay ID is C_341729_10; rs2336219, G>A, assay ID is C_16204465_10), *MLH3* (rs108621, T>C, assay ID is C_2178406_10) and *hOGG1* (rs1052133, G>C, assay ID is C_3095552_1).

### 2.3 Tumour chemosensitivity assay

To evaluate the effect of CDDP on the viability of primary lung cancer cells from NSCLC patients, MTT(3‐[4,5‐dimethylthiazol‐2‐yl]‐2,5‐diphenyl tetrazolium bromide) assay was performed to detect individual chemosensitivity to CDDP. Firstly, fresh tumour tissues were collected in surgery and immediately washed with the normal saline containing 1% Penicillin–Streptomycin. After the necrotic part was removed, the clean part was cut into mud in Dulbecco's Modified Eagle's Medium/Nutrient Mixture F‐12 (Gibco, Grand Island, NY, USA). After passing the mixture through a 125 μm nylon membrane, the flow‐through was centrifuged at 1000 *g* for 3 min to acquire single scattered cells, which were then cultured in a 96‐well plate. Subsequently, the cells were incubated for 24 h and treated with CDDP of 0, 0.5, 1, 2, 4, 8, 16, 30 μg/ml for another 24 h. Then, the culture plates were incubated with 20 μl of MTT/well in 100 μl medium at a final concentration of 5 mg/ml for another 4 h. Afterwards, the supernatant was carefully removed and 100 μl dimethyl sulfoxide was added to each well to discontinue the reaction. Besides, blue‐violet formazan particles were dissolved for 10 min in the dark at 25°C. Finally, the absorbance at 490 nm was detected by quant universal microplate spectrophotometer (BioTek Instruments, Inc.), and the sensitivity of each patient to CDDP was evaluated.

### 2.4 Cell culture and treatment

HEK293T and A549 cells were purchased from the Cell Bank of Shanghai institute of Biochemistry and Cell Biology, Chinese Academy of Sciences, and cultured in DMEM (Hyclone, USA) and DMEM/F‐12 (Hyclone, USA), respectively, which were supplemented with 10% foetal bovine serum (Hyclone, USA). These cells were maintained in a humidified incubator at 37°C with 5% CO_2_ and involved in experiments in the logarithmic growth stage. At the beginning and ending of the whole experiment, these cell lines were validated using STR authentication by Genetic Testing Biotechnology Co Ltd. (Suzhou, China). Besides, in order to prevent cross‐contamination during the experiments, each cell line operated independently. Additionally, the detection of mycoplasma in cell culture medium was performed with MycoSEQ™ Mycoplasma Detection Assay (Applied Biosystems, USA) every 2 weeks to ensure no contamination of mycoplasma in cells.

### 2.5 RNA extraction and real‐time qPCR

Total RNA was extracted by the TransZol™ Up Plus RNA Kit (TRANS, China), and followed by reverse transcription to acquire cDNA. Afterwards, the cDNA samples were sent to the Light Cycler 480 Real‐time PCR system (Roche, USA) to be amplified. In brief, the qPCR reaction program included 30 cycles of denaturation at 94°C for 30 s, annealing at 56°C (58°C for GAPDH) for 30 s and extension at 72°C for 30 s. Primers for qPCR were as follows: *ERCC1*, F:5'–GACGCCATCAACACCGAGTT–3′ and R:5'–CTTTGTCGTTGGTTAGCTGGT–3′; *GAPDH*, F: 5'–TGTGGGCATCAATGGATTTGG–3′ and R: 5'–ACACCATGTATTCCGGGTCAAT–3′. Finally, 2^−ΔΔCt^ method was adopted to quantify the relative expression of genes and *GAPDH* gene acted as an internal reference.

### 2.6 Western blot

Tumour tissue homogenate was made by tissue homogenizer before it was lysed in RIPA buffer solution. After a series of extraction operations following the manufacturer's instructions, 30–60 μg of total protein was collected for the detection of ERCC1 expression was acquired. Western blot was performed strictly according to the standard protocol and finally the immunoreactive bands were developed with hypersensitive chemiluminescence reagents (Beyotime, China). The relevant antibody information was as follows: ERCC1 (Abcam, ab129267, 1:1000) and β‐actin (ZSGB‐BIO, China, 1:5000), in which the latter acted as an internal reference.

### 2.7 Immunohistochemical staining

The procedure of immunohistochemical staining for ERCC1 expression was as follows. The tumour tissue samples from 22 NSCLC patients were sliced into 4 μm sections and the sequential operations including antigen repair, breaking endogenous peroxidase activities with 3% hydrogen peroxide, blocking non‐specific binding sites with 3% normal goat serum were performed. Afterwards, the sections were incubated with the ERCC1 (dilution 1:200, ab129267) antibody and the biotin‐labelled secondary goat anti‐rabbit immunoglobulin G (IgG), respectively, and then stained with diaminobenzidine and counter stained with haematoxylin. Finally, the slides were scanned, and the images were captured in five randomly selected visual fields on each slide for analysis under a Digital Pathology Scanner (Aperio CS2, Leica Biosystems, USA). The intensity of the dye colour was graded as 0 (no colour), 1 (light yellow), 2 (light brown) or 3 (brown), and the number of positive cells was graded as 0 (<5%), 1 (5%–25%), 2 (25%–50%), 3 (51%–75%) or 4 (>75%). The two grades were multiplied together, and specimens were assigned to one of 4 levels: 0–1 score (−), 2–4 scores (+), 5–8 scores (++), more than 9 scores (+++).

### 2.8 Dual‐luciferase reporter assay

Above all, bioinformatics analysis was conducted to acquire genomic information including the Targetscan database (http://www.targetscan.org/) and RNAhybrid database (https://bibiserv.cebitec.uni‐bielefeld.de/rnahybrid), from which we got the binding fragment of miRNA in *ERCC1* 3'UTR, the GenBank database (https://www.ncbi.nlm.nih.gov/genbank/), from which the upstream and downstream sequences of target genes were obtained and the Vector NTI software, by which relevant primers were favourably designed. Further, luciferase reporters were constructed for wild‐type (WT) and mutant (MUT) miRNA binding site of *ERCC1* rs3212986. The design and construction of mutant *ERCC1* were completed by Takara company (Dalian, China) and the corresponding plasmid sequences were as follows: *ERCC1*‐WT: 5'–ACAGGCTGCTGCTGCTGCTGCTTCCGCTTCTTGTCCCGGCCT–3′, *ERCC1*‐MT: 5'–ACAGGCACGACGACGACGACGAAGGCGAAGAACAGGGGGCCT–3′, and *ERCC1* rs3212986‐MUT: 5'–ACAGGCTGCTGCTGCTGCTTCTTCCGCTTCTTGTCCCGGCCT–3′. Next, the above artificially constructed *ERCC1* 3'UTR sequences were amplified before being cloned into pMIR‐REPORT vector containing a synthetic firefly luciferase gene which was specifically designed to be an intra‐plasmid transfection normalization reporter (Obio, China). Afterwards, HEK293T cells were seeded into 96‐well plates with 70% confluence and 24 h later transfected with miR‐15a mimics (100 nM, Qiagen, USA), miR‐4298 mimics (100 nM, Qiagen, USA) or negative control (NC) mimics (100 nM, Qiagen, USA), respectively, and simultaneously the above reporters used Lipofectamine™ 3000 Reagent (Invitrogen, USA). Finally, the cell extracts were prepared 48 h after transfection and the luciferase activity was measured by the Dual‐Luciferase Reporter Assay (Beyotime, China). Moreover, the firefly luciferase activity was normalized to the Renilla luciferase activity to derive the relative luciferase activity. The independent experiments were performed in triplicate.

### 2.9 Genotyping lung adenocarcinoma cell line A549

The genotype of rs3212986 polymorphism in A549 cells was detected and the allele‐specific primers synthesized via standard phosphor amidite synthesis were as follows: F: 5'–CACGAGCCCTTCTTGAAA–3′ and R: 5'–GAGCCAATTCAGCCACTA–3′. Besides, bidirectional DNA sequencing was performed at the Sangon Biotech and the sequence analysis was performed with Chromas 1.62 software (Helens vale, Queensland, Australia).

### 2.10 Transfection of miR‐15a mimics

A549 cells in the logarithmic phase were cultured for 24 h and then transfected with miR‐15a mimics (Qiagen, USA) or NC mimics (Qiagen, USA) with HiPerFect Reagent (Qiagen, USA) according to the manufacturer's protocol. After transfection for 48 and 72 h, the mRNA and protein levels of ERCC1 were detected, respectively. The total concentration of mimics in each case was kept constantly at 50 nM.

### 2.11 Detection of candidate miRNA expression

In brief, total RNA from tissues or cells was extracted using the miRNeasy Mini kit (Qiagen, Inc.), followed by the synthesis of cDNA with the miScript II RT kit (Qiagen, USA). Afterwards, the cDNA was amplified with miScript SYBR Green PCR kit (Qiagen, USA) in Light Cycler 480. The PCR reaction program included: 15 min at 95°C, 45 cycles of denaturation at 94°C for 15 s, annealing at 55°C for 30 s and extension at 70°C for 30 s. Primers for miR‐15a, miR‐4298 and U6 were purchased from Qiagen, and the relative expression levels of miRNAs were normalized to U6.

### 2.12 Statistical analysis

All data were statistically processed with spss19.0 software (SPSS, Inc.) and graphpad prism6.0 software (GraphPad Software, Inc.). χ^2^ test was used to analyse the association between the candidate polymorphisms and clinic‐pathological characteristics. Kaplan–Meier curves and log‐rank test were applied to assess the effect of candidate polymorphisms on overall survival (time between diagnosis and death or last follow‐up). In addition, Cox proportional hazard model was adopted to further clarify the association of candidate polymorphisms with overall survival after a preliminary univariate regression analysis. Corresponding hazard ratios and 95% confident intervals (95% CIs) were estimated after adjusting for age, gender, stage, pack year of smoking and treatment regimens. A two‐tailed *p*‐value <0.05 was considered statistically significant.

## 3 RESULTS

### 3.1 Association between the clinicopathologic characteristic and overall survival in NSCLC patients

This study incorporated 246 patients with NSCLC who received curative‐intent therapy, including surgical resection and platinum‐based chemotherapy. The average age was 59.3 years old, 62% of which are males. Most of them were adenocarcinomas (65.1%) and 78% were in early stage (I and II) (see Table S1). The median follow‐up period was 29 months (range: 8–65 months). Kaplan–Meier curves and log‐rank test were used to assess the association of the clinicopathologic characteristic with the overall survival. Our results demonstrated a significant difference among group of gender, tumour size, TNM stage and differentiation (*p* < 0.01). However, the overall survival did not show statistical significance in the group of lymphatic metastases (*p* = 0.09). Therefore, the above statistically significant variables in the univariate analysis finally entered the Cox proportional hazard model, including the lymphatic metastases due to their clinical relevance.

### 3.2 *ERCC1* rs3212986 AA genotype suggested an unfavourable prognosis in NSCLC patients

DNA repair mechanisms, including BER, NER and MMR, are of great significance to the repair of platinum‐DNA adducts. Accordingly, several relevant rate‐limiting enzyme genes in the three DNA repair pathways were selected in this study, namely hOGG1, ERCC1 and MHL3, respectively. After adjusting for the variables of gender, lymphatic metastasis, tumour size, TNM stage and differentiation, no statistical significance was found between the SNPs in *MLH3*, *hOGG1* genes and overall survival in the univariate analysis, while three SNPs of *ERCC1* including the rs735482, rs2336219 and rs3212986 exhibited different survival indices among different genotypes. Interestingly, when these three SNPs of *ERCC1* were incorporated into the multivariate Cox model, only rs3212986 polymorphism showed a clear statistical significance (Table 1).

**TABLE 1:** Association of polymorphisms with the overall survival analysed by Cox proportional hazards regression analysis

Gene	Variable	Univariate Cox			Multivariate Cox		
		Hazard ratio	95% CI	*p*‐value	Hazard ratio	95% CI	*p*‐value
*ERCC1*	rs1007616						
	TT						
	TC	0.169	1.184	0.544			
	CC	0.059	1.061	0.831			
	rs735482						
	CC						
	CA	−0.098	0.907	0.527	0.231	1.260	0.810
	AA	−0.474	0.622	0.019*	0.403	1.496	0.677
	rs2336219						
	GG						
	GA	−0.106	0.90	0.495	−0.261	0.770	0.786
	AA	−0.504	0.604	0.015*	−0.796	0.451	0.415
	rs3212986						
	CC						
	CA	0.234	1.264	0.111	0.053	1.054	0.746
	AA	0.655	1.925	0.005*	0.573	1.773	0.036*
*MLH3*	rs108621						
	TT						
	TC	0.081	1.084	0.587			
	CC	0.110	1.116	0.719			
*hOGG1*	rs1052133						
	CC						
	CG	0.147	1.159	0.45			
	GG	−0.059	0.943	0.772			

*Note*: Adjust by Gender, Lymphatic metastasis, Tumour size, TNM staging, Chemotherapy, Differentiation. *p < 0.05.

**FIGURE 1:**
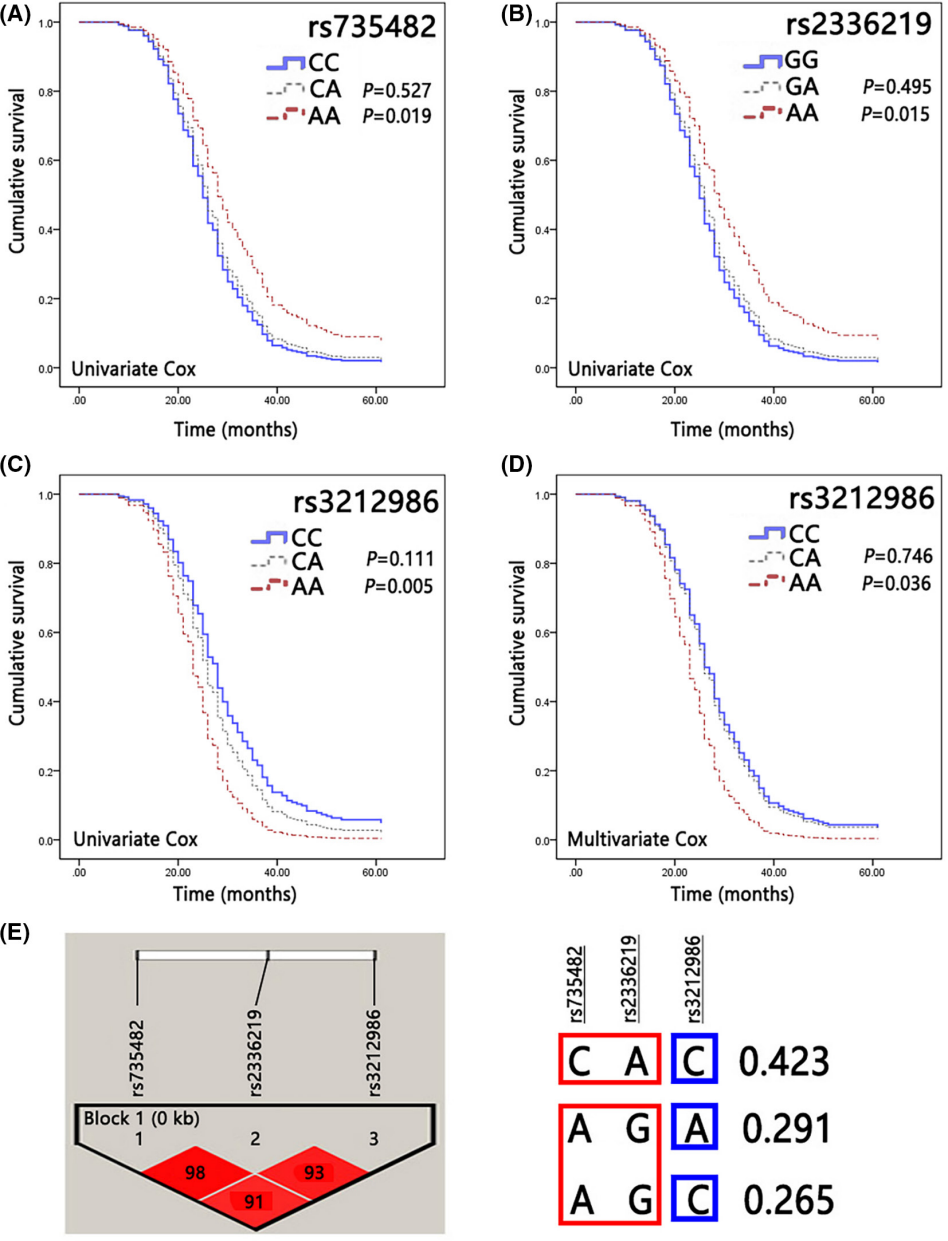
Association between candidate SNPs of *ERCC1* and overall survival of NSCLC patients. Based on Cox regression model, Gender, Lymphatic metastasis, Tumor size, TNM staging, Chemotherapy and differentiation were used as covariable, and the genotypes were analyzed as a prognostic variable. The value of SNPs including rs735482 (A) rs2336219 (B) and rs3212986 (C) in predicting survival was observed separately. All selected SNPs with significant differences were incorporated into COX model and Kaplan–Meier survival curves were shown for patients with NSCLC according to *ERCC1* rs3212986 polymorphisms (D). *p* < 0.05 was considered to indicate a statistically significant difference. Short dashed line: CC genotype group; solid line: CA genotype group. solid line: wild genotype group; Long dotted line: heterozygous genotype group; Short dotted line: mutant genotype group. (E) Based on the analysis of linkage disequilibrium about the sequence polymorphisms of *ERCC1* gene, strong LD structure was identified through the entire region of this gene.

The univariate analysis showed that the patients carrying AA genotype of rs735482 had a significantly higher survival index than those with CC genotype (Figure 1A; *p* < 0.05). Meanwhile, the median survival time of the patients carrying AA genotype of rs2336219 was apparently longer than that of those with GG genotype (Figure 1B; *p* < 0.05). However, the multivariate Cox model did not show that rs735482 and rs2336219 had any effect on survival indices. Instead, the patients carrying AA genotype of rs3212986 were associated with a significantly shorter survival time in both univariate (Figure 1C; *p* < 0.05) and multivariate analyses (Figure 1D; *p* < 0.05). Based on the above results, we further analysed the linkage disequilibrium of rs735482, rs2336219 and rs3212986 and found a strong linkage imbalance between C allele of rs735482 and A allele of rs2336219 (D′: 0.423), and between A allele of rs735482 and G allele of rs2336219 (D′: 0.291, 0.265) (Figure 1E). Interestingly, the alleles with linkage imbalance showed an opposite effect on the overall survival (the CC genotype of rs735482 for negative survival indices vs the AA genotype of rs2336219 for positive ones; the AA genotype of rs735482 for positive survival indices vs the GG genotype of rs2336219 for negative ones) (Figure 1A,B), which might partly explain why these two SNPs lost their prognostic value in the multivariate Cox model. In a nutshell, our data supported the hypothesis that *ERCC1* rs3212986 polymorphism might be an independent predictor for the prognosis of NSCLC patients receiving platinum‐based chemotherapy.

### 3.3 *ERCC1* rs3212986 AA genotype was linked with a higher expression of *ERCC1* and a lower sensitivity to cisplatin

**FIGURE 2:**
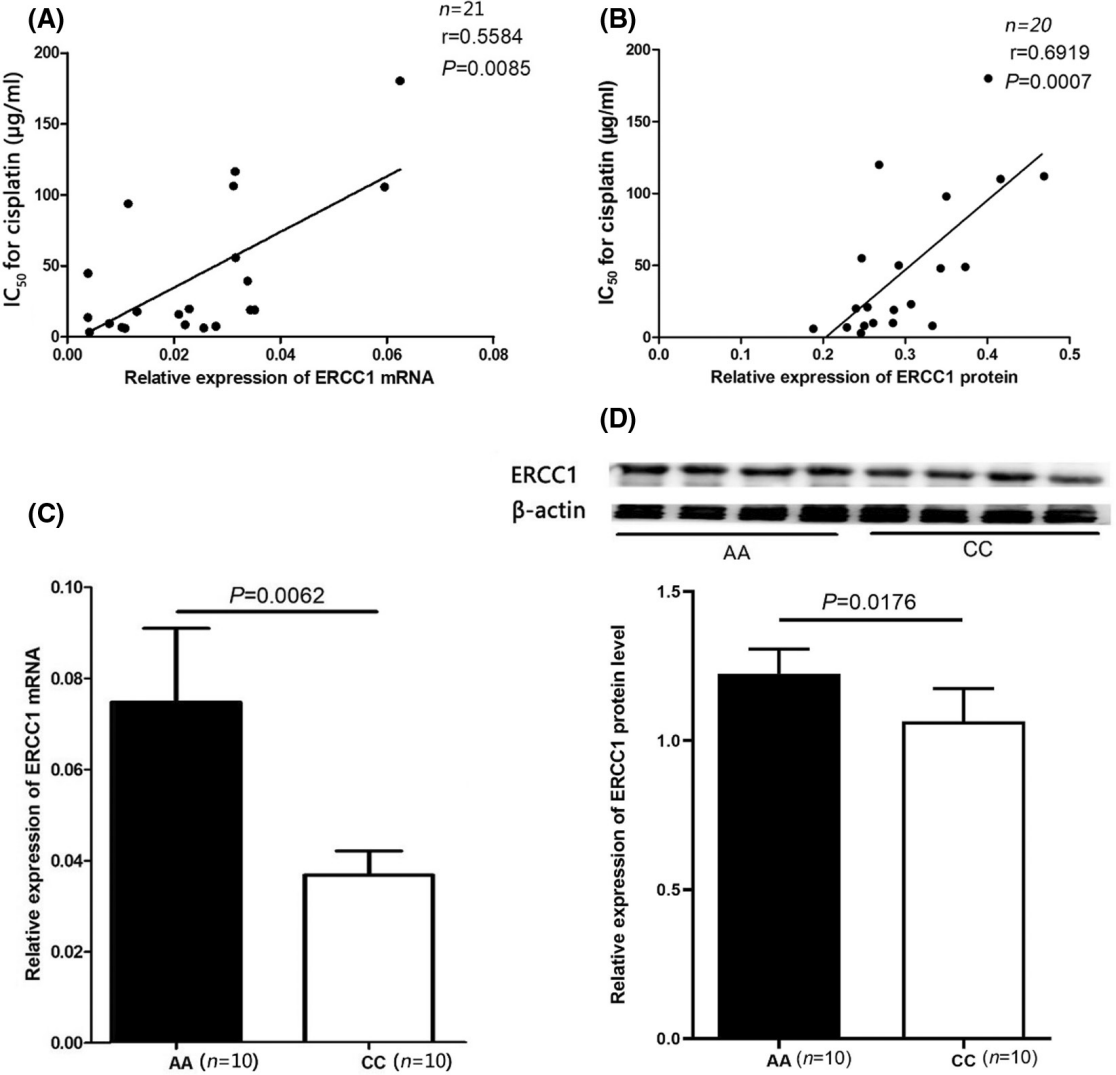
ERCC1 expression linked with CDDP sensitivity and compared in the AA and CC genotype of rs3212986 polymorphism. Association of *ERCC1* mRNA (A) and protein (B) expression with the sensitivity to CDDP of tumor cells was analyzed. Comparison of *ERCC1* mRNA (C) and protein (D) in tumor tissues by rs3212986 genotypes. Protein levels of ERCC1 were quantified by image j software.

In order to verify the clinical relevance of *ERCC1* rs3212986 polymorphism, the primary tumour cells from NSCLC patients were cultured and attacked with CDDP to evaluate the individual sensitivity to platinum analogues. The IC_50_ values provided by the MTT assay showed a wide discrepancy of cells in response to cisplatin treatment. Consistent with previous reports, whether the high expression of ERCC1 mRNA or protein was positively correlated with the resistance of tumour cells to CDDP (Figure 2A,B). Furthermore, the mRNA and protein expression of ERCC1 in tumour tissues were compared between the AA and CC genotypes of rs3212986. The data from RT‐qPCR and Western Blot, respectively, indicated that the average levels of *ERCC1* mRNA and protein in patients carrying AA genotype was significantly higher than those with CC genotype (Figure 2C,D). Therefore, we speculated that rs3212986 polymorphism was linked with a different sensitivity to cisplatin via altering the expression of *ERCC1*.

### 3.4 MiR‐15a was screened and verified as a target miRNA binding to rs3212986

**FIGURE 3:**
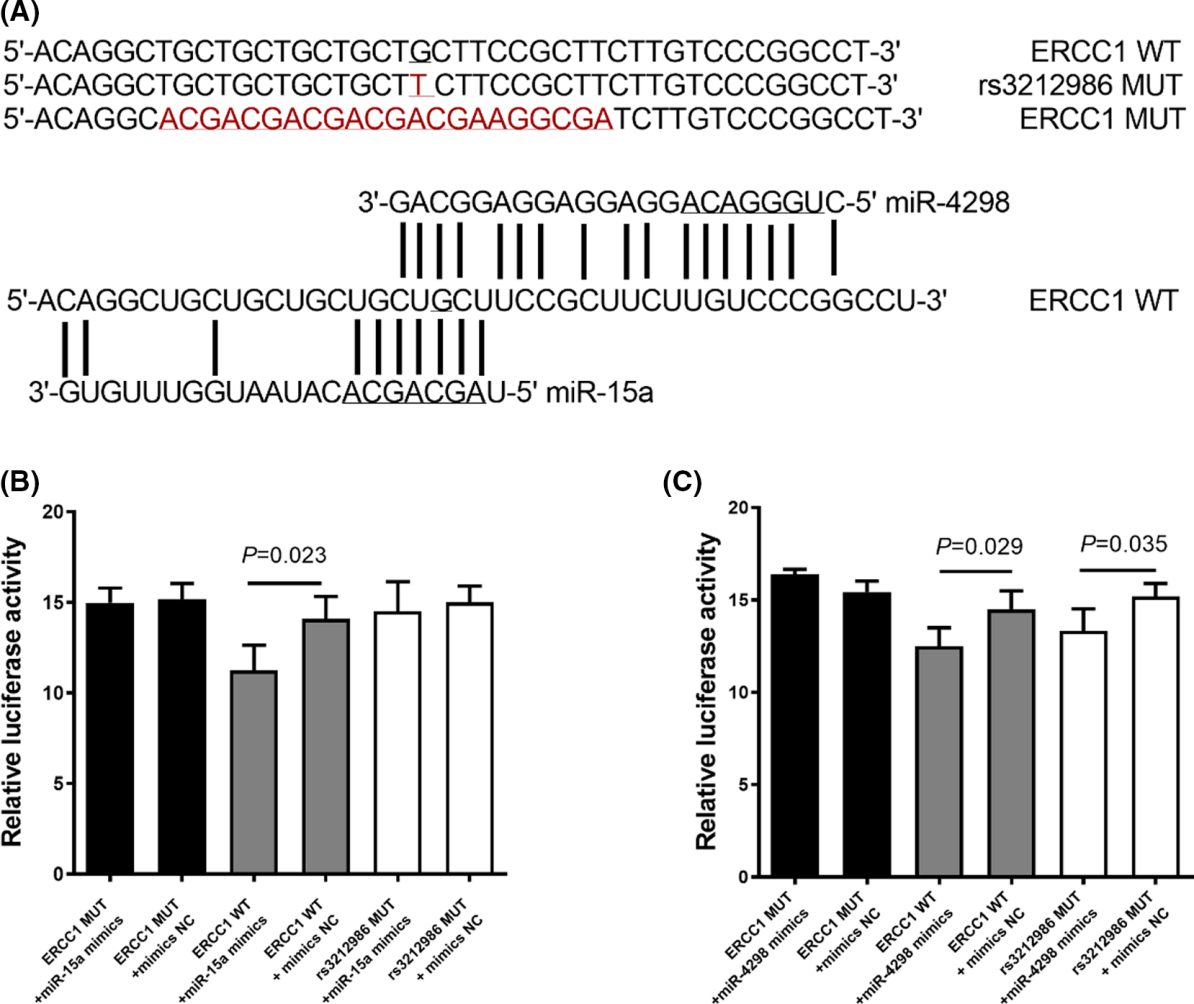
Direct binding of miR‐15a and miR‐4298 to *ERCC1* 3'UTR. Rs3212986 polymorphism was located on the seed sequence of miR‐15a binding to *ERCC1*. The structure diagram of miR‐15a binding to *ERCC1* 3'UTR was predicted by Targetscan, and the sequence of rs3212986 MUT, *ERCC1* MUT and WT was used for the dual luciferase reporter assay. The relative luciferase activity was measured in HEK293T cells after co‐transfection of the *ERCC1* luciferase construct with either miR‐15a or control (A). In HEK293T cells, the suppression of luciferase activity was only apparent when miR‐15a was partnered with wild‐type CC (B), while miR‐4298 significantly inhibited the luciferase activity, whether mutated or not (C).

We presumed that the presence of some miRNAs can affect the post‐transcriptional regulation of ERCC1 and alter its expression by binding to 3'UTR. Accordingly, the potential miRNAs binding to *ERCC1* rs3212986 were predicted by the Targetscan and RNAhybrid databases. Finally, two miRNAs including the miR‐15a and miR‐4298 were identified, and the minimum free energies were −40.80 Kcal/mol for miR‐4298 and −25.80 Kcal/mol for miR‐15a, respectively (see Table S2). The above results indicated that *ERCC1* 3'UTR contains potential target sequences for miR‐15a and miR‐4298, and rs3212986 located in the MRE (miRNA response element) of mature miR‐15a (Figure 3A). To evaluate the direct effects of miR‐15a and miR‐4298 on *ERCC1* expression, we constructed luciferase reporter plasmids containing the WT or MUT 3'‐UTR of *ERCC1* (including the *ERCC1* MUT and rs3212986 MUT), and then co‐transfected them into HEK293T cells with miR‐15a mimics, miR‐4298 mimics or NC mimics, respectively. Interestingly, miR‐15a significantly suppressed the luciferase activity in the *ERCC1* WT group (*p* < 0.05) (Figure 3B) but did not show any effect in the rs3212986 MUT group. However, a down‐regulated luciferase activity was found in both *ERCC1* WT (*p* < 0.05) and rs3212986 MUT groups (*p* < 0.05) after the transfection of miR‐4298 (Figure 3C). Besides, co‐transfection of miRNA mimics and *ERCC1* MUT reporter plasmids showed no effect on luciferase activity. Our data demonstrated that although the luciferase activity could be regulated by both miR‐15a and miR‐4298, only miR‐15a‐mediated regulation of *ERCC1* expression was diminished when rs3212986 C allele was transferred to A allele.

### 3.5 MiR‐15a decreased *ERCC1* expression in A549 cells carrying rs3212986 CC genotype

**FIGURE 4:**
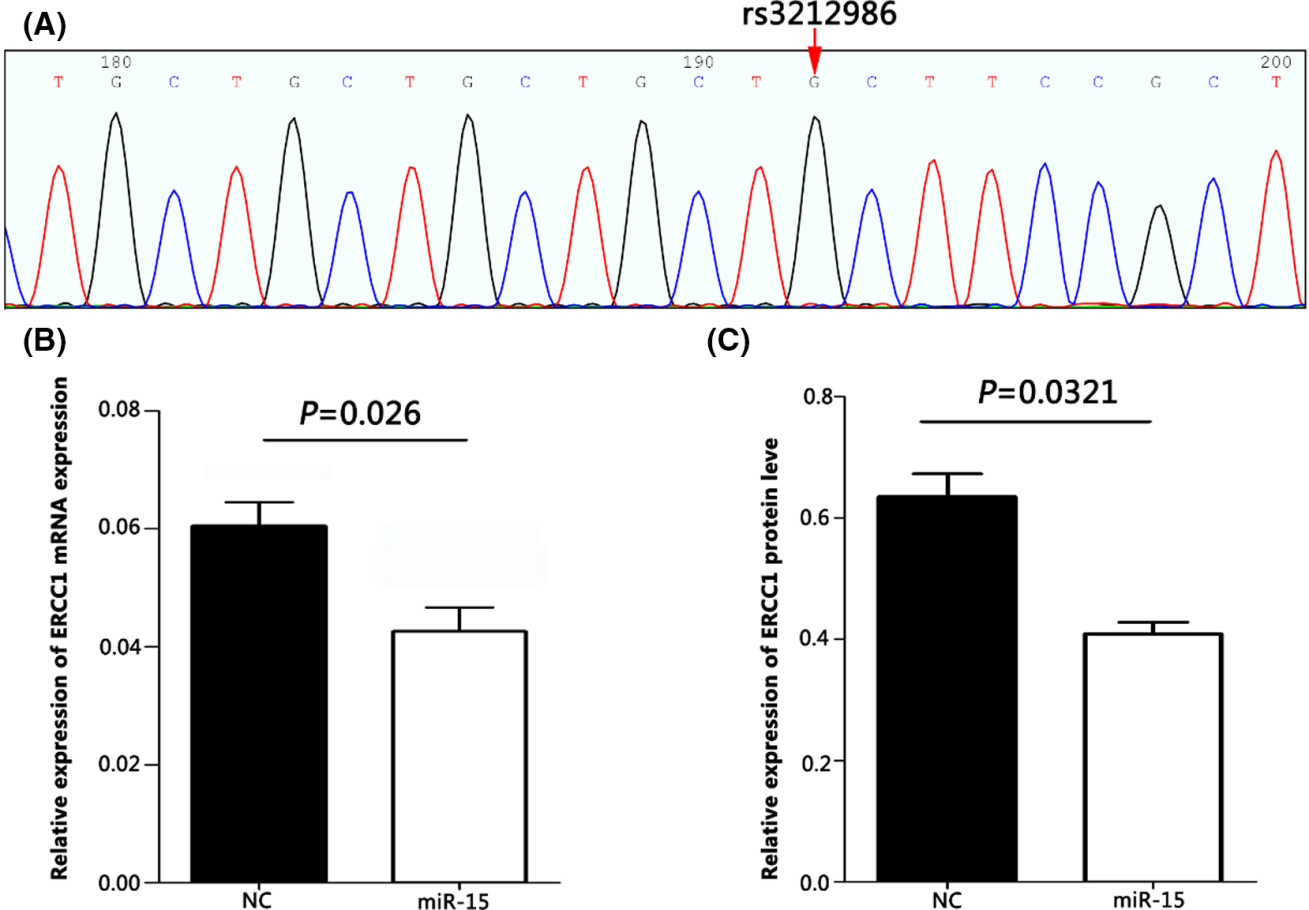
MiR‐15a decreased ERCC1 expression in A549 cells. Plot of Sanger Sequencing of A549 cell, the nucleotide referred to G (A). *ERCC1* mRNA levels in A549 cell transfected with miR‐15a or mimics NC were analyzed by qRT‐PCR and normalized to GAPDH (B). Western blot analysis of ERCC1 protein in A549 cells transfected with miR‐15a or mimics NC (C).

Sanger sequencing results of human lung adenocarcinoma cell line A549 was presented in Figure 4A, in which the SNP site of rs3212986 only showed a single G product peak, indicating that 549 cells carried the CC genotype of rs3212986. Subsequently, the A549 cells were transfected with miR‐15a to further confirm the effects of miR‐15a on the expression of *ERCC1* in the context of CC genotype of rs3212986. The results showed that miR‐15a indeed down‐regulated the expression of *ERCC1* whether in mRNA or protein (Figure 4B,C), suggesting miR‐15a might have a causal relationship with the lower expression of ERCC1 and the higher sensitivity to cisplatin in those carrying rs3212986 CC homozygote.

### 3.6 *ERCC1* expression was negatively linked with miR‐15a in NSCLC tissues carrying rs3212986 CC genotype

**FIGURE 5:**
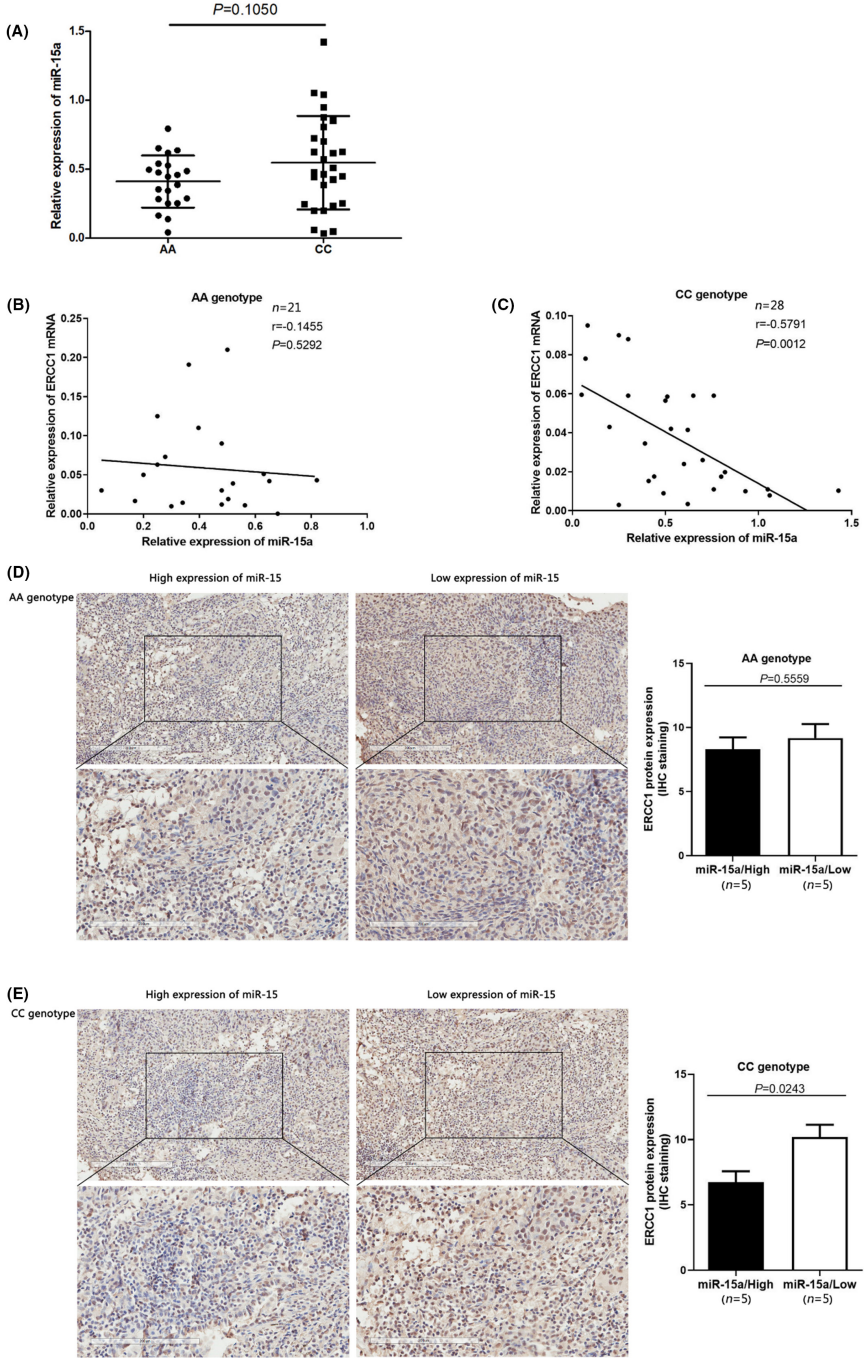
*ERCC1* expression was negatively correlated with miR‐15a expression exclusively in tumor tissues carrying rs3212986 CC genotype. Expression of miR‐15a in NSCLC tissues carrying different rs3212986 genotypes (A). Correlation between the expression of miR‐15a and *ERCC1* mRNA in NSCLC tissues carrying AA (B) and CC (C) genotypes. U6 was used as an internal control to normalize the expression of miR‐15a. Correlation between ERCC1 protein and the expression of miR‐15a in NSCLC tissues carrying AA (D) and CC (E) genotype.

To verify the correlation between the *ERCC1* rs3212986 polymorphism and miR‐15a, the expression of miR‐15a was firstly detected in tumour tissues carrying different genotypes of rs3212986 and no difference was observed between the two genotypes (Figure 5A), which suggested that the rs3212986 polymorphism had no effect on the expression of miR‐15a. However, the expression of miR‐15a was negatively correlated with the *ERCC1* mRNA expression in tumour tissues carrying CC genotype, but not in AA one (Figure 5B,C). Considering the post‐transcriptional regulation of miR‐15a on *ERCC1* mRNA, we further performed an immunohistochemical staining on clinical tumour tissues to determine the correlation between the expression of ERCC1 protein and miR‐15a. As shown in Figure 5D,E in tumour tissues with CC genotype, the low expression of ERCC1 was generally observed in the high expression group of miR‐15a (E), but not in AA genotype (D). Based on the analysis here, our data suggested that ERCC1 expression might be affected by rs3212986 polymorphism via the regulation of miR‐15a, which helps to partially explain the higher sensitivity to platinum‐based chemotherapeutics in NSCLC patients carrying rs3212986 CC genotype due to a lower expression level of ERCC1 mediated by miR‐15a.

## 4 DISCUSSION

As known, the pharmacological role of platinum‐based chemotherapeutics is to form platinum‐DNA adducts, which will lead to the inhibition of proliferation in tumour cells. In this case, the DNA repair capacity in tumour cells has an immense impact on clinical efficacy in NSCLC patients who receive platinum‐based chemotherapeutics.^30^, ^31^ ERCC1 as a highly conserved structure‐specific endonuclease, functions in the NER pathway and cuts the damaged strand from 5′ to the site of damage^32^ and its polymorphisms have been widely studied and gradually regarded as biomarkers to predict the risk and prognosis of multiple cancers. Our previous study has also demonstrated that the minor allele in rs3212986 polymorphism was related to a higher level of BPDE‐DNA adducts induced by benzopyrene,^33^ and the A allele of rs3212986 reflected a linkage with the elevated risk of NSCLC.^34^ Accordingly, whether rs3212986, the potential clinically relevant polymorphism was involved in the prognosis of NSCLC remained to be further clarified. A potential effect of *ERCC1* rs3212986 polymorphism on the overall survival of NSCLC patients was proposed by our data, which suggested that the patients carrying rs3212986 A allele were associated with a poorer response to platinum‐based chemotherapy and a shorter survival time in contrast to those carrying C allele, consistently with the previous reports.^35^, ^36^

The rs3212986 polymorphism showed a close relationship with the overall survival and prognosis in advanced NSCLC patients treated with platinum‐based chemotherapy.^37^, ^38^ On the contrary, rs735482 and rs2336219 polymorphisms could not be determined as an independent biomarker to predict the benefits from platinum‐based chemotherapy because there was a linkage imbalance in their alleles which reminded the opposite survival indices. Interestingly, this may partially explain the interactions of the two SNPs loci in simultaneous analysis and their functions may offset each other beyond our imagination. Overall, our data provided some supportive evidence that genetic variations could interact with each other, thus emphasizing the necessity of exploring a causal relationship.

Considering the importance of the post‐transcriptional regulation of *ERCC1*, the rs3212986 located in *ERCC1* 3' UTR might lead to allele‐specific change in the binding of some certain miRNAs, so the following mechanistic studies should focus on the effects of specific miRNAs. In fact, some studies suggested that the dysregulation of miRNAs has been indicated as an alternative mechanism of platinum resistance,^39^ and miRNA mimics as an alleviator of drug resistance have been verified to alter the gene expression via miRNA‐mRNA interactions.^40^ Besides, several deregulated miRNAs have been reported in radiation and chemo‐resistance of lung cancer,^41^, ^42^ suggesting that miRNAs could be promising targets to improve the response to chemotherapy.

In this study, some candidate miRNA, including miR‐15a and miR‐4298, were screened and predicted by bioinformatics. These two miRNAs were bound to *ERCC1* rs3212986, which was only located at the MRE binding site of mature miR‐15a. As known, miR‐15a was one of the first described miRNAs associated with cancers.^43^ Based on the prediction results of computational models, miR‐15a was identified to be associated with lung neoplasms.^44^ Moreover, several papers recently have demonstrated that miR‐15a acted as a tumour suppressor, suggesting its potential effect on the prognosis of NSCLC patients.^45^, ^46^, ^47^, ^48^ However, the discussion on causal association between miR‐15a and NSCLC was defective in the previous studies. Our in vitro functional assays put forward that miR‐15a acted as an enhancer of the sensitivity of NSCLC cells to cisplatin, which might contribute to elevating the benefits of platinum‐based chemotherapy in patients carrying rs3212986 C allele via altering the post‐transcriptional regulation of *ERCC1*. However, *ERCC1* could not be effectively regulated by miR‐15a in those carrying rs3212986 A allele. So, we believe that it might be partly explained by the miRNA‐mediated post‐transcriptional regulation on ERCC1. In fact, it has been reported that, in addition to inhibiting protein expression at the translation level, miRNAs could also affect mRNA stability by promoting its degradation.^49^, ^50^, ^51^, ^52^, ^53^ Our current study also confirmed that ERCC1 mRNA could be degraded after the transfection of miR‐15a mimics, which suggested in the context of rs3212986 CC genotype, both ERCC1 mRNA and protein could be regulated by miR‐15a and it might be one of the mechanisms leading to the lower mRNA and protein of ERCC1 in CC genotype. Consequently, our study might provide new evidence that the rs3212986 polymorphism may affect the individual sensitivity of platinum‐based chemotherapy by altering ERCC1 expression.

Although the exact mechanism has not been completely clarified, and a larger sample size is also necessary to confirm the findings of clinical investigation, our study integrated the survival analysis of NSCLC patients with in vitro functional exploration and partly explained that rs3212986 polymorphism might be linked with the sensitivity to platinum analogues via affecting the post‐transcriptional regulation of ERCC1 and alter the DNA repair capacity of tumour cells. Moreover, increasing studies have been devoted to developing computational models to predict potential miRNA‐disease associations.^54^, ^55^ Then, the accumulation of biological data, the combination between the depth computational prediction model and causal experimental verification could form an effective method to explore miRNA‐disease associations.

**FIGURE 6:**
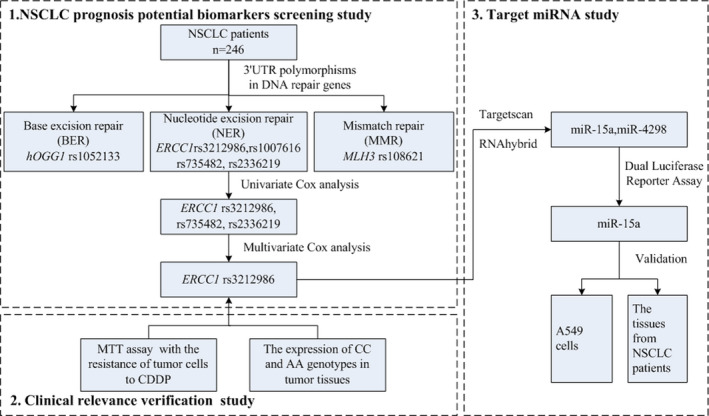
The basic process and main findings of the present study

Briefly, the basic process and main findings of this study were summarized and shown in Figure 6.

## 5 CONCLUSION

Rs3212986, a 3'UTR polymorphism in *ERCC1*, is linked with the sensitivity to platinum analogues via altering ERCC1 expression due to the binding of miR‐15a. As a potential prognostic biomarker, the rs3212986 polymorphism is expected not only to help clinicians perform personalized chemotherapy based on different genetic backgrounds of patients (e.g. the patients with AA genotype of rs3212986 may not be suitable for platinum‐based chemotherapy) but also to contribute to making more accurate prediction for the prognosis of NSCLC patients who receive platinum‐based chemotherapy.

## AUTHOR CONTRIBUTIONS

**Ping Xue:** Data curation (equal); formal analysis (equal); writing – original draft (equal); writing – review and editing (equal). **Guopei Zhang:** Data curation (equal); formal analysis (equal); writing – original draft (equal); writing – review and editing (equal). **Hongchao Zhang:** Data curation (equal); formal analysis (equal); writing – original draft (equal); writing – review and editing (equal). **Su Cui:** Investigation (equal). **Liang Zhang:** Investigation (equal). **Tao Yu:** Data curation (equal). **Mingyang Xiao:** Data curation (equal); formal analysis (equal). **Liuli Li:** Data curation (equal); formal analysis (equal). **Xiaobo Lu:** Funding acquisition (lead); methodology (lead); project administration (lead); resources (lead); supervision (lead); writing – review and editing (equal).

## CONFLICT OF INTEREST

The authors declare that they have no known competing financial interests or personal relationships that could have appeared to influence the work reported in this paper.

## Supporting information


Table S1
Table S2

## Data Availability

The data used to support the findings of this study are included within the article.
