# Supplementary material for: A miR‐15a related polymorphism affects NSCLC prognosis via altering ERCC1 repair to platinum‐based chemotherapy
Source: J Cell Mol Med. 2022 Sep 30;26(21):5439–51. doi: 10.1111/jcmm.17566 (PMC9639052; doi:10.1111/jcmm.17566)
Supplement: Supplementary file 1 — Table S1 Table S2 [file JCMM-26-5439-s001.docx]

**Supplementary Table S1** Characteristic of patients with non-small cell lung cancer and the effect on overall survival

| Factors | All patients (n = 246) | Log Rank χ^2^ value | *P* |
| --- | --- | --- | --- |
| Age (y) |  |  |  |
| <60 | 122 |  |  |
| ≥60 | 124 | 0.433 | 0.511 |
| Gender |  |  |  |
| Male | 153 |  |  |
| Female | 93 | 6.597 | 0.01^a^ |
| Smoking |  |  |  |
| No | 132 |  |  |
| Yes | 114 | 0.172 | 0.679 |
| Alcohol consumption |  |  |  |
| No | 126 |  |  |
| Yes | 120 | 0.25 | 0.617 |
| Tumor size |  |  |  |
| <3cm | 102 |  |  |
| ≥3cm | 144 | 4.956 | 0.026^a^ |
| Lymphatic metastasis |  |  |  |
| No | 124 |  |  |
| Yes | 122 | 2.877 | 0.09^a^ |
| TNM staging |  |  |  |
| Ⅰ+Ⅱ | 192 |  |  |
| Ⅲ+Ⅳ | 54 | 12.525 | <0.001^a^ |
| Differentiation |  |  |  |
| low-grade (G 3) | 86 |  |  |
| high-grade (G 1+ G 2) | 160 | 45.935 | <0.001^a^ |

a: Variables included in further Cox regression Model.

**Supplementary Table S2** The predicted miRNAs binding to *ERCC1* 3’UTR

| miRNAs^a^ | Binding Position of *ERCC1* 3’UTR(bp)^b^ | minimum free energy(mfe) (kcal/mol) ^c^ | structure diagram of miRNA binding to *ERCC1* 3’UTR^d^ |
| --- | --- | --- | --- |
| miR-15a | 176-199 | -25.8 | 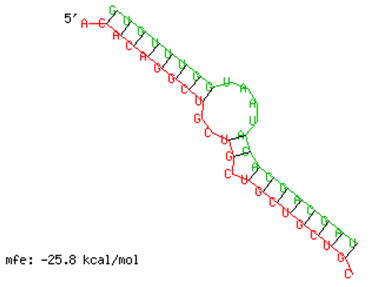 |
| miR-4298 | 194-216 | -40.8 | 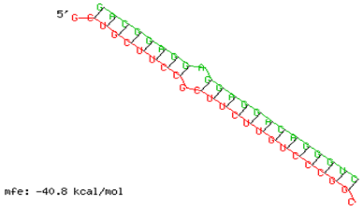 |

a: The target miRNAs of *ERCC1* 3’UTR were predicted using Targetscan and RNAhybrid combining with the published research related to lung cancer. b: *ERCC1* rs3212986 located in 197bp of *ERCC1* 3’UTR. c: The miRNA response element of miRNA binding to *ERCC1* 3’UTR was predicted by RNAhybrid, which was the most likely binding position preferentially. d: The structure diagram of miRNA binding to *ERCC1* 3’UTR was predicted by RNAhybrid.
